# A Preclinical Porcine Model of Portal Vein Thrombosis in Liver Cirrhosis

**DOI:** 10.1155/2020/3086906

**Published:** 2020-04-08

**Authors:** Rui Zhang, Shenxin Lu, Ying-yi Jiang, Jing-qin Ma, Wen Zhang, Jun-ying Gu, Jian Wang, Shi-yao Chen

**Affiliations:** ^1^Department of Gastroenterology and Hepatology, Zhongshan Hospital, Fudan University, Shanghai, China; ^2^Shanghai Institute of Liver Disease, Shanghai, China; ^3^Liver Cancer Institute, Zhongshan Hospital, Fudan University and Key Laboratory of Carcinogenesis and Cancer Invasion, Ministry of Education, Shanghai, China; ^4^Department of Interventional Radiology, Zhongshan Hospital, Fudan University, Shanghai, China; ^5^Department of Radiology, Zhongshan Hospital, Shanghai, China; ^6^Department of Medical Imaging, Shanghai Medical College, Fudan University, Shanghai, China; ^7^Endoscopy Center and Endoscopy Research Institute, Zhongshan Hospital, Fudan University, Shanghai, China

## Abstract

**Background:**

This study aimed at presenting a novel method of developing a porcine model of portal vein thrombosis (PVT) in cirrhosis by intravenous administration of thrombin and insertion of a fibered coil. We further investigated changes of biochemical parameters, coagulation, and proinflammatory cytokine expression in the cirrhosis-PVT group.

**Methods:**

Twelve male pigs were randomized into the control group (*n* = 3) and cirrhosis group (*n* = 9). In cirrhotic pigs, three were randomly selected to establish PVT by ultrasound-guided percutaneous puncture of the main portal vein (MPV) followed by intravenous thrombin administration and fibered coil insertion. Thrombosis in the MPV was detected by abdominal enhanced computer tomography (CT). The changes of hepatic function, coagulation system, and inflammation cytokines were compared among normal, cirrhosis, and cirrhosis with PVT groups.

**Results:**

As manifested by the presence of a filling defect in MPV on portal venous-phase CT angiography, fibrin thrombi were formed in the MPV in cirrhotic pigs within one week and persisted for four weeks. Five weeks after surgery, abnormal liver functions occurred in association with PVT formation in cirrhosis. Both coagulation and thromboelastography parameters showed that cirrhosis-PVT pigs exhibited a procoagulant state through hyperfunction of platelets and clotting factors. Interleukin 6 (IL-6) as a potential inflammatory marker stimulated PVT-mediated inflammation activation in cirrhosis.

**Conclusions:**

Our study provides *in vivo* evidence that intravenous injection of a coil and thrombin into MPV under interventional guided devices enables a feasible method in thrombus creation. Further exploration and validation of large-sample cases are required to characterize utilities of this model.

## 1. Introduction

Portal vein thrombosis (PVT) is defined as the presence of thrombosis within the main portal vein (MPV) with or without extension to intrahepatic branches [1]. Approximately 20% of patients with cirrhosis are complicated by PVT [2]. As the degree of thrombosis progresses from partial to complete, the presentation of PVT ranges from asymptomatic signs to severe complications such as variceal bleeding and portal hypertension (PHT), which may become a technical contraindication for liver transplantation (LT) or negatively impact the survival outcomes [3, 4]. On the other hand, although the guidelines accepted the anticoagulant treatments or transjugular intrahepatic portosystemic shunt (TIPS) as a therapeutic option for PVT in cirrhosis, PVT with cavernous transformation usually resulted in the failure of TIPS [5]. The technical success of TIPS and patients' survival also closely depended on the degree of MPV occlusion [6]. Further, the present literatures do not establish with certainty the role of antithrombotic agents in preventing PVT extension in cirrhosis patients [7].

Several evidences have suggested that PVT is a disease with multifactorial causes, but the exact physiopathological mechanisms leading to PVT in cirrhosis remained to be fully deciphered. In some cases, it has been recognized that the components of Virchow's triad were the main factors involved in PVT development in cirrhosis patients. In other cases, PVT in cirrhosis was triggered by a genetic predisposition and inflammation effects [8–11]. Previous techniques have successfully induced animal models of PVT, PHT, and vena thrombosis [12–15]. However, the established animal model of PVT in cirrhosis remains an unsolved issue to date.

In view of the current clinical situation and the lack of adequate basic research of PVT in cirrhosis, we constructed a pig model of cirrhosis with PVT and performed preliminary comparisons in healthy control, cirrhosis, and cirrhosis with PVT groups for the evaluation of biochemical and coagulant parameters and systemic inflammation.

## 2. Materials and Methods

### 2.1. Animal Models of Cirrhosis

Male pigs (each weighing 10~12 kg of body weight, Yorkshire strains) aged one and a half month were purchased from Jiagan Corporation (Shanghai, China). A certain degree of fibrosis was achieved prior to PVT induction. In the cirrhosis group, pigs were subjected to carbon tetrachloride (CCl_4_) (*n* = 9). CCl_4_ in 50% olive oil was intraperitoneally injected at a dosage of 0.25 mL/kg twice weekly and continued for 12 weeks [16]. To determine cirrhosis induction, one pig was sacrificed at the 12th week for liver pathology. The control group comprised pigs that received olive oil (*n* = 3). To prevent spontaneous regression of cirrhosis, CCl_4_ injections were continued biweekly throughout the study in cirrhosis with and without PVT groups. Finally, all cirrhotic pigs with or without PVT were pathologically confirmed. All animals were housed and treated humanely in accordance with the protocols outlined by the Shanghai Medical Experimental Animal Care Commission (Shanghai, China). The ethical guideline was approved by the Zhongshan Hospital Research Ethics Committee (Shanghai, China).

### 2.2. Thrombosis Model in the Portal Vein of Cirrhosis

At 12 weeks of CCl_4_ injection, construction of the thrombosis model was randomly carried out in 3 male cirrhotic pigs. The animals were fasted for 12 h preoperative procedures and then underwent general anesthesia with a mixture of intramuscular injection of 15 mg/kg of xylazine hydrochloride (Jilin, China) and 10 mg/kg of diazepam (Tianjin, China).

PVT was induced using a combination of a fibered coil import (MWCE-35-14-8-NESTER, Cook Incorporated, Bloomington, USA) and intravenous injection of recombinant thrombin (Sangon, Shanghai, China). Briefly, the abdominal skin of the pigs kept in a supine position was sterilized. Using a color Doppler ultrasound diagnostic instrument, a 4F catheter sheath (Merit Medical System Inc., USA) was advanced into the right branch of PV over a 0.035-inch J-shaped guidewire with a movable core (Cook, USA) following a successful puncture of PV branches with an 18 G puncture needle. Next, a 4F VER catheter (Cordis, USA) was advanced through the catheter sheath into the MPV. Preliminary portal venography was performed by injecting 12–18 mL of iopromide (Bayer) into the MPV to evaluate the status of venous blood flow and ensure that the catheter head was in the MPV. Then, an 8 mm diameter coil (Cook) was delivered to the MPV, followed by the slow administration of 1000 U thrombin dissolved in 10 mL of saline to the central of MPV proximal to the coil. Again, venography of postthrombus induction was repeated by placing the catheter in the MPV to observe the position of the coil and the blood flow of the portal vein, and assess the lack of contrast opacification of the MPV or its branches. Finally, the catheter was removed.

### 2.3. Laboratory Measurements and Thromboelastography

Five weeks after thrombus operation, femoral venous blood samples of pigs in control, cirrhosis, and cirrhosis-PVT groups were collected for hematologic parameter testing of hepatic and coagulation functions following the standard procedures. Biochemical analyses and coagulation screening tests were measured with the spectrophotometric analysis and the turbidimetric method, respectively. Thrombelastography (TEG) was performed using Thrombelastograph Hemostasis Analyzer System (Haemonetics Corporation, USA). Thromboelastographic parameters consisted of clot time (*R* time, the time from the start of sample tracing till the first TEG trace amplitude reaching 2 mm), clot formation time (*K* time, the time from the measurement of *R* to the formation of fixed clot firmness), rate of clot formation (*α* angle, the speed of fibrin buildup and crosslinking), maximum amplitude (MA, the maximum amplitude on the TEG tracing), and coagulation index (CI, derived from *R*, *K*, *α* angle, and MA). Herein, *R* denotes the rate of initial fibrin formation, *K* represents the clot kinetics, *α* angle is the fibrinogen level, MA reflects the strength of the clot, and CI describes the overall coagulation. The serum levels of interleukin 6 (IL-6), IL-8, and tumor necrosis factor alpha (TNF-*α*) were detected with a chemiluminescent analyzer (IMMULITE 1000 Immunoassay System, Siemens Healthineers, Germany).

### 2.4. Histopathological Analysis

All specimens were fixed with 10% formalin buffer, embedded in paraffin, and cut into 4 to 5 *μ*m sections. The degree of fibrosis, liver collagen fibers, and thrombus formation in the portal vein were examined using hematoxylin and eosin and Masson's trichrome staining. All images were photographed at 200x magnification under a microscope (Olympus BX51, Tokyo, Japan).

### 2.5. Transmission Electron Microscopy Analysis

Platelet aggregation in the hepatic sinusoidal vessels was captured using transmission electron microscopy. Briefly, liver sections were harvested and immediately fixed using 2.5% glutaraldehyde and 0.1 mol/L sodium cacodylate. Next, liver samples were dehydrated using ethanol and propylene oxide. The specimens were embedded in Araldite, cut into ultrathin sections at 50–60 nm, and stained with 3% lead citrate-uranyl acetate. All images were acquired using a JEM-1200 electron microscope.

### 2.6. Contrast-Enhanced Abdominal Computed Tomography Imaging Evaluation of PVT

A contrast-enhanced abdominal CT scanner was applied as a monitoring system for PVT formation in cirrhosis. After general anesthesia, the pig was placed in the supine or lateral decubitus position followed by the 64-slice spiral computed tomography (CT) examination (Siemens, Germany). A single abdominal CT scan was performed first according to the scanning protocols and parameters (slice thickness of 5 mm, weight-based tube potential of 120 kVp, automatic tube current modulation of 75–500 mA, 0.5 s gantry rotation time, 5 mm layer spacing, pitch of 0.85, moving speed at 5–10 m/s, and 6 to 9 s scanning time). Further, 70 mL of intravenous contrast medium (iodide, 3 mg/mL) was administered through a pressure injector at a rate of 3 mL/s. This performance was operated by a technician with 20 years of experience. All the CT examinations were reevaluated by a radiologist with more than 7 years of experience in interpreting abdominal CT examination.

### 2.7. Statistical Analysis

All continuous variables were expressed as mean ± standard deviation and statistically analyzed using the GraphPad Prism 6 Software (San Diego, CA, USA). Statistical comparisons for parameters of hepatic function, coagulation function, thromboelastography, and systemic inflammation between two groups (cirrhosis versus control, cirrhosis with PVT versus control, and cirrhosis with PVT versus cirrhosis) were separately made using an unpaired Student *t*-test. A *P* value less than 0.05 was defined as statistical significance.

## 3. Results

### 3.1. Baseline Characteristics of Animals

A total of 12 male pigs were included in our experiment, and all of them were randomly assigned into control (*n* = 3) and cirrhosis groups (*n* = 9). In the cirrhosis group, 2 pigs were excluded due to death related to acute severe liver injury and 1 pig was sacrificed for liver pathology. Of the remaining 6 cirrhotic pigs, 3 were randomly selected and subjected to PVT construction. During the experimental period, loss of appetite and tarnished hair tended to appear in pigs of cirrhosis and cirrhosis with PVT groups. At the end of the experiment (17 weeks later), the body weight of pigs in the cirrhosis and cirrhosis with PVT groups significantly decreased compared to that in the healthy control group (43.33 ± 2.49 vs. 52.67 ± 2.05 kg, *P* = 0.015; 42.33 ± 2.49 vs. 52.67 ± 2.05 kg, *P* = 0.011). Nevertheless, there was no considerable difference in the body weight between the cirrhosis group and the cirrhosis with PVT group (43.33 ± 2.49 vs. 42.33 ± 2.49 kg, *P* = 0.71).

### 3.2. Detection of PVT in Cirrhosis

**Figure 1:**
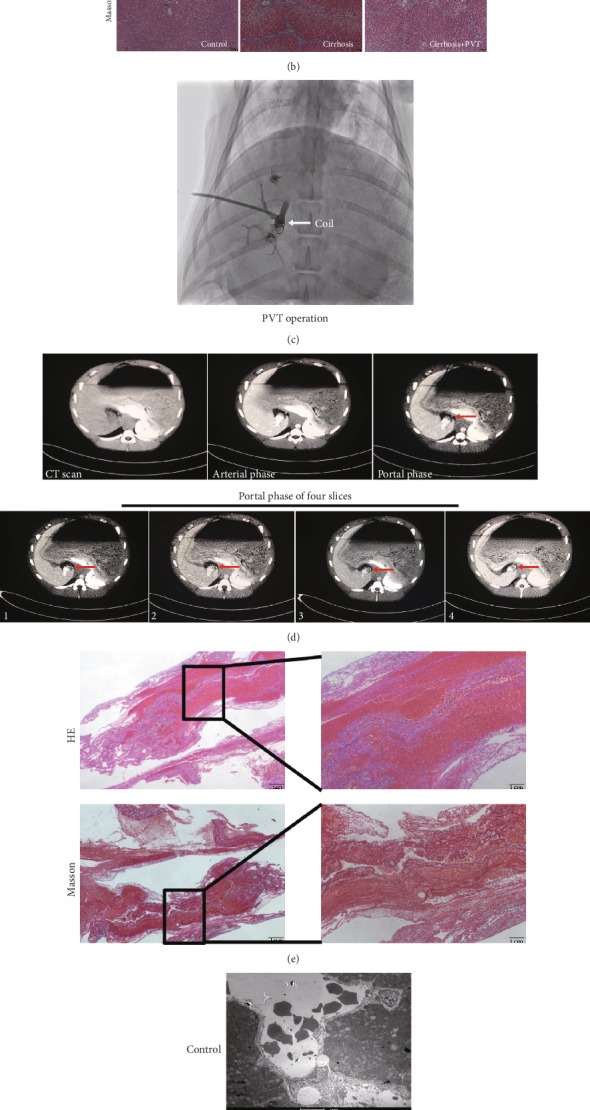
PVT in a cirrhotic porcine model was successfully induced by the placement of a fibered coil into the main portal vein through a sheath introducer followed by the intravenous thrombin administration. (a) General features of representative liver specimens in healthy and cirrhotic pig models. (b) Representative image presenting pathological changes of the porcine model in normal, cirrhosis, and cirrhosis with PVT groups. (c) One representative image of PVT induction in cirrhosis. (d) CT examination of a filling defect on a portal venous phase showed the formation of thrombosis. (e) Organized thrombi adjacent to the intima of the portal vein. (f) Platelet aggregation (white arrow) adjoining the vessel in cirrhotic liver specimens was identified.

Three months later, the liver texture was soft, smooth, and reddish brown in the normal control group, while the liver of the cirrhosis group was hard and shrunken with the formation of grey nodules (Figure 1(a)). Histopathological results demonstrated that hepatic sinus was in spoke and polygon distribution in the control group throughout the study. By contrast, gross fibrous tracts and extensive pseudolobular formation were demonstrated in the pig model of cirrhosis with and without PVT groups (Figure 1(b)). In the PVT model, percutaneous intravenous thrombin administration and fibered coil insertion were utilized to establish PVT (Figure 1(c)). It was found that PVT was formed in cirrhosis pigs within one week. The PVT follow-up term was 5 weeks after interventional surgical operation. As demonstrated by contrast-enhanced CT examination, a partially obstructive thrombus in the main portal vein (MPV) was exemplified by the presence of a filling defect on portal venous-phase CT (Figure 1(d)). Histologically, organized and fibrin-rich thrombi adjacent to the intima of MPV were also observed (Figure 1(e)). Together, results of the present findings confirmed the successful establishment of PVT in cirrhosis.

### 3.3. Liver Dysfunction and Prothrombotic State in Pigs with Cirrhosis with and without PVT

There was marked increase in serum levels of ALT, AST, FIB, and D-dimer in cirrhotic pigs versus healthy control (Table 1). Histochemistry morphometric analysis identified platelet aggregation near the vessels in liver specimens in the cirrhosis group (Figure 1(f)). Furthermore, serum levels of ALT, AST, TBIL, CHE, PT, APTT, INR, FIB, and D-dimer were further elevated, and serum ALB levels were further lowered in cirrhosis-PVT pigs compared to normal and cirrhotic pigs (Table 1). TEG results indicated that TEG-R significantly decreased in the cirrhosis with and without PVT groups compared with the normal group. While TEG-*α*, TEG-MA, and TEG-CI were markedly increased in the cirrhosis with PVT group compared with the non-PVT and healthy control groups (Table 2). Collectively, these results supported that liver and coagulation dysfunction initially occurred in cirrhosis. PVT further worsened the liver function and increased thrombotic risk of cirrhosis.

### 3.4. Increased Levels of Serum IL-6 in Cirrhosis with and without PVT

It has been demonstrated that elevation of systemic inflammation contributed to increased risk of chronic liver disease and venous thromboembolism [17–19]. Subsequently, serum inflammatory cytokines were detected in our animal models. As illustrated in Table 3, levels of IL-6 were significantly increased in cirrhosis and cirrhosis-PVT pigs in comparison with the control group. In addition, circulating IL-6 in cirrhosis with PVT was further elevated compared to that in cirrhosis with non-PVT pigs, whereas nonsignificant alterations were identified as regards serum IL-8 and TNF-*α* levels. Overall, these findings suggested that IL-6 may be responsible for the proinflammatory effects as a consequence of an improvement by PVT in cirrhosis.

## 4. Discussion

PVT is a frequent complication of cirrhosis, and its prevalence increases with the progression of cirrhosis. In addition, PVT also caused exacerbation of PHT, resulting in complications of ascites and gastrointestinal bleeding, which worsen the quality of life of cirrhosis patients [20, 21]. Our preclinical study aimed at developing a cirrhosis-PVT model in a large animal model (pig) that has CCL4-induced cirrhosis and depicting the changes of hepatic function, coagulation system, and inflammation cytokines. The described technique represents a new animal model, with high applicability to investigation of the mechanism and pathophysiological effects of PVT on cirrhosis development, and the benefit of anticoagulant treatment in promoting thrombosis recanalization in cirrhosis-PVT. As previously suggested [22], PVT is also generally recognized as a prognostic factor in hepatocellular carcinoma (HCC). The HCC patient population with Child-Pugh B in the setting of PVT has been difficult to treat. This preclinical model and the experimental method may also have a significant impact on the preclinical evaluation of locoregional HCC therapies and provide further benefit research in this area.

Previously, creating an animal model of PVT, PHT, or vena thrombosis has been successfully attempted [12–15]. However, no effective models of cirrhosis with PVT have been introduced. Here, we firstly described the placement of a coil insertion into the MPV through a sheath introducer followed by the intravenous thrombin administration. The manifestation of a filling defect on portal venous-phase CT after modeling favored the evidence of thrombus in the MPV. Then, it was revealed that cirrhotic pigs with PVT were associated with worse liver function and prothrombotic state, as proven by significant improvements of ALT, AST, ALB, TBIL, PT, APTT, INR, FIB, and D-dimer, and alteration of thromboelastographic variables. TEG is an established clinical laboratory method to simulate the dynamic changes of coagulation, solidification, and fibrinolysis. It also allows for quantitatively determining whether the body environment has a hypercoagulable state, low coagulation, fibrinolysis, or other symptoms. Based on the scope of TEG parameters, personalized approaches to guide antithrombotic therapies could be determined [23–25]. Our TEG results revealed that the level of TEG-R was prominently decreased and levels of TEG-MA, TEG-CI, and TEG-*α* were significantly increased, indicating that the activation of plasma clotting factors and platelet function might account for the enhanced coagulation in cirrhosis with PVT. While the evidence of liver inflammation contributing to progressive liver disease can be derived from the previous study [19, 26], the explanation for the role of PVT in the inflammatory changes in cirrhotic environment has not been specifically studied. This *in vivo* study further demonstrated that elevated IL-6 level exerted a critical role in cirrhosis and thrombosis promotion. Targeting anticoagulation and anti-inflammation (IL-6) may be promising therapeutic strategies for cirrhosis with PVT. Our findings were in agreement with the previous findings of our reports in human cirrhosis patients with PVT [27].

The method of thrombus formation was speculated to be either surgical procedures including flow restriction (electrolytic stasis, stasis, stenosis, and ligation) or vessel injury (mechanical trauma) [28–33]. Disadvantages were that most experiments were traumatically performed on rodents and the thrombi were rapidly dissolved. In addition, the development of PVT has been restrained by a more analogous animal model. Pigs are large animals very similar to humans in anatomy, physiology, and pathology, making them an extremely appropriate model to create PVT. Compared with surgical methods, the interventional technology is minimally invasive and convenient for observation. In the vessel, thrombin led to the formation of a platelet/fibrin-rich thrombus [34]. Not only is nester coil originally intended for arterial and venous embolization but it also conforms inside the vessel to create a tight occluding mass [35–37]. Therefore, combining a fibered coil and thrombin in cirrhotic pigs were reasonably and feasibly attempted for PVT induction. Additionally, the maintained time for PVT can be sustained for at least 4 weeks. In a clinical scenario, PVT formation in cirrhosis is multifactorial, and therefore, it is beneficial to perform in-depth studies from a model only by slowing portal vein flow as it can exclude other confounding factors.

The study had several limitations. Firstly, our current study might be affected by the relatively small number of animals, especially whether the statistical evaluations for parameters of systemic inflammation are significant in each comparison. Further validation in a large cohort should be performed. Secondly, the combination of a fibered coil import and intravenous thrombin injection is not a completely physiological state and may disturb the secondary development of collaterals. Thirdly, the model was only a preliminary investigation. The underlying mechanism by which abnormal hepatic function, inflammation activation, and the elevation of IL-6 level were induced directly by PVT establishment, aggravating cirrhosis or both needed to be demonstrated in additional studies.

We conclude that a combination of thrombin and coil insertion holds promise as an effective and safe method to induce PVT in a cirrhotic pig model. The development of this model has a closer analogy to clinical practice and is of great importance in providing platforms for thrombosis-related evaluations.

## Figures and Tables

**Table 1:** Animal characteristics in the control, cirrhosis, and cirrhosis with PVT groups.

Laboratory variables	Control	Cirrhosis	Cirrhosis with PVT
ALT (U/L)	43.00 ± 2.16	72.17 ± 0.85^a^	80.67 ± 3.30^b,c^
AST (U/L)	29.17 ± 1.65	50.67 ± 1.25^a^	97.67 ± 2.05^b,c^
ALB (g/L)	34.53 ± 0.41	33.47 ± 0.41	29.67 ± 1.25^b,c^
TBIL (*μ*mol/L)	0.65 ± 0.04	0.74 ± 0.04	1.15 ± 0.04^b,c^
CHE (U/L)	113.67 ± 53.90	183.67 ± 9.84	248.67 ± 20.30^b,c^
PT (s)	9.50 ± 0.16	9.77 ± 0.09	11.03 ± 0.05^b,c^
APTT (s)	13.43 ± 0.33	13.67 ± 0.62	21.70 ± 0.80^b,c^
INR	0.83 ± 0.02	0.82 ± 0.02	0.96 ± 0.005^b,c^
FIB (mg/dL)	122.0 ± 2.94	153.33 ± 3.86^a^	176.33 ± 7.13^b,c^
D-dimer (mg/L)	1.90 ± 0.16	3.28 ± 0.19^a^	5.02 ± 0.12^b,c^

ALT: alanine aminotransferase; AST: aspartate aminotransferase; ALB: albumin; TBIL: total bilirubin; CHE: cholinesterase; PT: prothrombin time; APTT: activated partial thromboplastin time; INR: international normalized ratio; FIB: fibrinogen level; PVT: portal vein thrombosis. ^a^*P* < 0.05 between the cirrhosis group and control group. ^b^*P* < 0.05 between the cirrhosis with PVT group and control group. ^c^*P* < 0.05 between the cirrhosis with PVT group and cirrhosis group.

**Table 2:** Thrombelastography variables in the comparison of control, cirrhosis, and cirrhosis with PVT.

TEG parameters	Control	Cirrhosis	Cirrhosis with PVT
TEG-CI	5.20 ± 0.16	5.83 ± 0.05^a^	6.33 ± 0.21^b,c^
TEG-K (min)	0.83 ± 0.05	0.77 ± 0.05	1.10 ± 0.08^b,c^
TEG-MA (mm)	75.53 ± 1.60	75.13 ± 0.26	80.80 ± 0.43^b,c^
TEG-R (min)	3.03 ± 0.21	2.07 ± 0.09^a^	1.87 ± 0.09^b^
TEG-*α* (deg)	74.67 ± 0.45	78.67 ± 0.62^a^	82.40 ± 1.10^b,c^

CI: coagulation index; *K*: coagulation time; MA: maximum amplitude; *R*: reaction time; *α*: alpha angle; PVT: portal vein thrombosis; TEG: thrombelastography. ^a^*P* < 0.05 between the cirrhosis group and control group. ^b^*P* < 0.05 between the cirrhosis with PVT group and control group. ^c^*P* < 0.05 between the cirrhosis with PVT group and cirrhosis group.

**Table 3:** Inflammatory cytokines in the comparison of control, cirrhosis, and cirrhosis with PVT.

Inflammatory cytokines	Control	Cirrhosis	Cirrhosis with PVT
TNF-*α* (pg/mL)	4.17 ± 0.24	4.10 ± 0.14	4.13 ± 0.19
IL-6 (pg/mL)	2.27 ± 0.09	8.27 ± 0.17^a^	23.07 ± 1.31^b,c^
IL-8 (pg/mL)	5.10 ± 0.14	5.17 ± 0.24	5.13 ± 0.19

IL-6: interleukin 6; IL-8: interleukin 8; TNF-*α*: tumor necrosis factor alpha; PVT: Portal vein thrombosis. ^a^*P* < 0.05 between the cirrhosis group and control group. ^b^*P* < 0.05 between the cirrhosis with PVT group and control group. ^c^*P* < 0.05 between the cirrhosis with PVT group and cirrhosis group.

## Data Availability

The data sets used in the study are available from the corresponding author on reasonable request.

## Abbreviations

PVT: Portal vein thrombosis
CC14: Carbon tetrachloride 4
PHT: Portal hypertension
LT: Liver transplantation
TIPS: Transjugular intrahepatic portosystemic shunt
PP: Portal pressure
HCC: Hepatocellular carcinoma.

## Additional Points

*Key Summary*. A combination of coil insertion and intravenous injection of thrombin into the MPV was a feasible method in thrombus creation. Cirrhosis-PVT pigs exhibited abnormal liver functions and procoagulant states. IL-6 as a potential inflammatory marker stimulated PVT-mediated inflammation activation in cirrhosis.
